# Membrane-enclosed multienzyme (MEME) synthesis of 2,7-anhydro-sialic acid derivatives

**DOI:** 10.1016/j.carres.2017.08.008

**Published:** 2017-11-08

**Authors:** Marie Monestier, Dimitrios Latousakis, Andrew Bell, Sandra Tribolo, Louise E. Tailford, Ian J. Colquhoun, Gwenaelle Le Gall, Hai Yu, Xi Chen, Martin Rejzek, Simone Dedola, Robert A. Field, Nathalie Juge

**Affiliations:** a Quadram Institute Bioscience, The Gut Health and Food Safety Institute Strategic Programme, Norwich Research Park, Norwich NR4 7UA, UK; b Department of Chemistry, University of California-Davis, Davis, CA 95616, USA; c Department of Biological Chemistry, John Innes Centre, Norwich Research Park, Norwich NR4 7UH, UK; d Iceni Diagnostic Ltd, The Innovation Centre, Norwich Research Park, Norwich NR4 7GJ, UK

**Keywords:** Sialic acid enzymatic synthesis, *Ruminococcus gnavus*, Intramolecular *trans*-sialidase, 2,7-anhydro-Neu5Ac, 2,7-anhydro-Neu5Gc

## Abstract

Naturally occurring 2,7-anhydro-alpha-N-acetylneuraminic acid (2,7-anhydro-Neu5Ac) is a transglycosylation product of bacterial intramolecular *trans*-sialidases (IT-sialidases). A facile one-pot two-enzyme approach has been established for the synthesis of 2,7-anhydro-sialic acid derivatives including those containing different sialic acid forms such as Neu5Ac and N-glycolylneuraminic acid (Neu5Gc). The approach is based on the use of *Ruminoccocus gnavus* IT-sialidase for the release of 2,7-anhydro-sialic acid from glycoproteins, and the conversion of free sialic acid by a sialic acid aldolase. This synthetic method, which is based on a membrane-enclosed enzymatic synthesis, can be performed on a preparative scale. Using fetuin as a substrate, high-yield and cost-effective production of 2,7-anhydro-Neu5Ac was obtained to high-purity. This method was also applied to the synthesis of 2,7-anhydro-Neu5Gc. The membrane-enclosed multienzyme (MEME) strategy reported here provides an efficient approach to produce a variety of sialic acid derivatives.

## Highlights

• A novel membrane enclosed multiple enzyme approach was developed for the production of 2,7-anhydro-sialic acid derivatives.
• 2,7-anhydro-Neu5Ac was produced in high-yield and high-purity.
• Neu5Gc-rich glycoprotein was produced using a one-pot two-step enzymatic synthesis.
• The method allowed the synthesis of a novel compound, 2,7-anhydro-Neu5Gc.

## Graphical abstract

Membrane-enclosed multienzyme (MEME) approach for the production of 2,7-anhydro-sialic acid derivatives.

**Figure:**
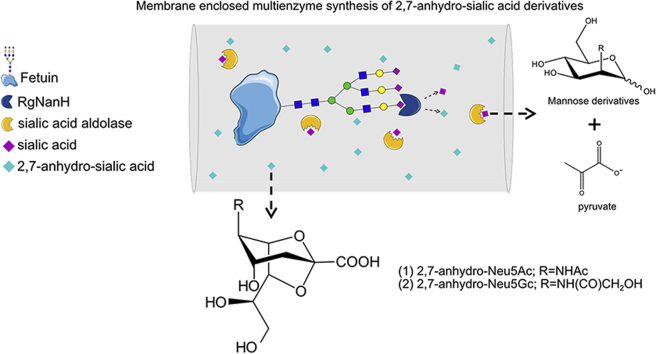

## 1 Introduction

Sialic acids constitute a structurally diverse family of nine-carbon acidic monosaccharides commonly found at the termini of the glycan chains on glycoproteins and glycolipids [1]. *N*-Acetylneuraminic acid (Neu5Ac), *N*-glycolylneuraminic acid (Neu5Gc), and 2-keto-3-deoxy-D-*glycero*-D-*galacto*-nonulosonic acid (KDN) are the three basic forms of sialic acids which are distinguished from one another by different substituents at carbon-5. Additional modifications include for example acetylation, lactylation, methylation, sulfation, resulting in more than 50 structurally distinct sialic acids [2]. As the outermost carbohydrate residues, sialic acids are critical recognition elements in a number of biologically important processes including cell-cell interaction, bacterial and viral infection, and tumor metastasis [3], [4]. For example, terminal sialic acid residues attached via α2, 3/6 glycosidic linkages to mucin glycan chains are prominent targets for commensal and pathogenic bacteria [5], [6], [7], [8].

**Fig. 1:**
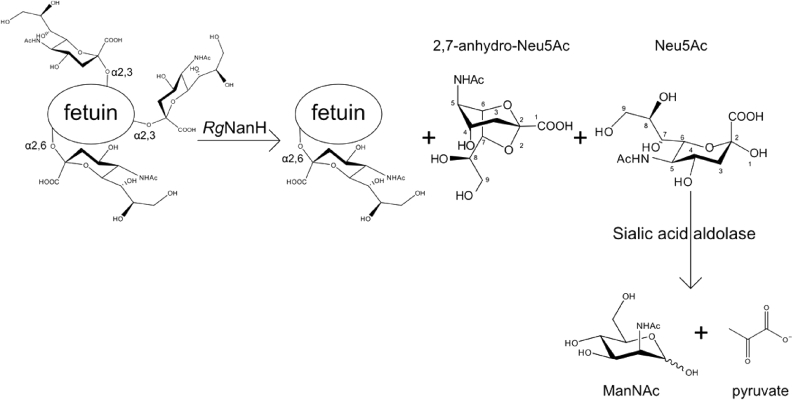
Overview of the reaction scheme. *Rg*NanH hydrolyses α2,3-linked-Neu5Ac on fetuin, releasing 2,7-anhydro-Neu5Ac as the major product but also Neu5Ac. Neu5Ac is further degraded into N-acetylmannosamine (ManNAc) and pyruvate by a sialic acid aldolase, which is inactive against 2,7-anhydro-Neu5Ac.

The release of sialic acid by microbial sialidases allows the bacteria present in the mucosal environment to access free sialic acid for catabolism, unmask host ligands for adherence, participate in biofilm formation, modulate immune function by metabolic incorporation, and expose the underlying glycans for further degradation [7], [8], [9], [10]. Most are hydrolytic sialidases, which release free sialic acid from sialylated substrates. However, there are also examples with transglycosylation activities. Intramolecular *trans*-sialidase (IT-sialidase), represents a new class of sialidases recently identified in pathogenic and commensal bacteria, releasing 2,7-anydro-N-acetylneuraminic acid (2,7-anhydro-Neu5Ac) instead of sialic acid [11], [12], [13]. Reaction specificity varies, with hydrolytic sialidases demonstrating broad activity against α2,3-, α2,6- and α2,8-linked substrates, whereas IT-sialidases appear specific for α2,3-linked substrates [7]. Recently, an IT-sialidase, *Rg*NanH, from the gut symbiont *Ruminoccocus gnavus* was structurally and functionally characterised. This enzyme produces 2,7-anhydro-Neu5Ac from α2,3-linked sialic acid glycoproteins or oligosaccharides [13], [14] (Fig. 1). 2,7-Anhydro-Neu5Ac was found to be a preferential metabolic substrate for *R. gnavus* strains expressing the IT-sialidase, suggesting a role in the adaptation of gut symbionts to the mucosal environment [15]. Previously, 2,7-anhydro-Neu5Ac had been detected in rat urine [16] and human wet cerumen (ear wax) [17]. It was suggested that this unusual sialic acid derivative may have bactericidal activity and/or could serve as a reservoir for sialic acids in the biological systems [17], [18].

At present, the biological significance of naturally occurring 2,7-anhydro-Neu5Ac in body fluid and secretions is still largely unknown. This is due to the lack of effective synthetic methods for its production. Lifely et al. [19] showed that methanolysis of sialic acid gave the methyl ester of 2,7-anhydro-Neu5Ac in addition to the methyl ester ketoside of sialic acid. The first directed synthesis of 2,7-anhydro-Neu5Ac was completed efficiently by intramolecular glycosidation of the *S*-(1-phenyl-1H-tetrazol-5-yl) glycoside derivative of Neu5Ac using silver triflate – bis(acetonitrile) palladium(II) chloride as a promoter [20]. More recently, the production of 2,7-anhydro-Neu5Ac was achieved using a leech IT-sialidase [21] and 4-methylumbelliferyl Neu5Ac (4MU-Neu5Ac) as substrate using sequential purification steps including precipitation, Folch partitioning and Bio-Gel purification [18].

Here we report a facile membrane-enclosed multienzyme (MEME) approach for the preparative synthesis of 2,7-anhydro-Neu5Ac (**1**) and 2,7-anhydro-Neu5Gc (**2**) from glycoproteins using *R. gnavus* IT-sialidase (*Rg*NanH) and a commercial sialic acid aldolase-catalysed reaction in one pot (see Fig. 1). The MEME synthesis offers several advantages over the existing synthetic methods such as the improved yield, the fact that the synthesis is scalable and that the route is greatly simplified (one step reaction). In addition, the MEME offers a cheaper solution to obtain sialic acid derivatives considering the cost of the starting material used in the previously reported chemical or enzymatic synthesis. The synthesis of 2,7-anhydro-Neu5Ac was achieved at high purity and in 20 mg scale using fetuin as substrate. The use of Neu5Gc-rich glycoproteins as donor substrates for preparing 2,7-anhydro-Neu5Gc is also described. Obtaining these compounds at a preparative scale is crucial for studying the biological importance of 2,7-anhydro-sialic acid derivatives in the gut and their potential applications in the biomedical sector.

## 2 Materials and methods

### 2.1 Materials

Fetuin from bovine serum, asialofetuin (type II), bovine submaxillary mucin, cytidine triphosphate, orcinol, CMP-sialic acid synthetase from *Neisseria meningitidis* group B, α2,3-sialyltransferase from *Pasteurella multocida,* ammonium formate and Dowex 1 × 8 anion exchange resin were purchased from Sigma Aldrich (St Louis, USA). 4MU-Neu5Ac is from Toronto Research Chemicals (ON, Canada). Sialic acid aldolase from *Escherichia coli*, 3′-sialyllactose (3′SL) and Neu5Gc were purchased from Carbosynth Limited (Compton, UK), BioGel P2 from Bio-Rad laboratories and SnakeSkin™ Dialysis Tubing, 7 kDa molecular weight cut off (MWCO), 22 mm from Thermo Fisher Scientific (Hemel Hempstead, UK). Nanopure water (18.2 MΩ cm; NanoPureThermo fisher Barnstead Water Reverse osmosis Purification System) was used for buffer preparation and for purification. *Rg*NanH was produced and purified as described previously [13]. 3′-glycolylneuraminyllactose (Neu5Gcα2-3Lac) was synthesised as previously reported [22].

### 2.2 Enzymatic synthesis of 2,7-anhydro-sialic acid

A dialysis membrane (7 kDa molecular weight cut off MWCO) containing fetuin, modified asialofetuin or bovine submaxillary mucin (15 mg/mL in 100 mM ammonium formate pH 6.5) was incubated in 100 mM ammonium formate pH 6.5 for 2 h at 37 °C with gentle shaking (115 rpm). Following the addition of 50 nM *Rg*NanH (in Tris buffer 40 mM, pH 7.9) into the dialysis membrane, the reaction mixture was incubated in a new solution of 100 mM ammonium formate (pH 6.5) at 115 rpm, 37 °C for 24 h. Then 0.5 units/mL of sialic acid aldolase (in 100 mM ammonium formate pH 6.5) was added to the membrane enclosed reaction mixture. Following a further 20 h incubation at 115 rpm and 37 °C, the dialysate (100 mL) was recovered, diluted with 100 mL of ultrapure water and freeze dried. The membrane enclosed reaction mixture was dialysed with 100 mL ultrapure water for 2 h and again overnight with 115 rpm shaking and at 37 °C. The water was recovered, pooled with the dried dialysate and freeze dried. After complete dryness, the powder was dissolved in 100 mL Nanopure water and freeze dried again 3 times to remove volatile salts.

### 2.3 Purification of 2,7-anhydro-sialic acid

The freeze-dried sample corresponding to crude 2,7-anhydro-Neu5Ac was dissolved in ultrapure water (2 mL) and purified by anion exchange chromatography using a Dowex 1 × 8 column. The anion exchange resin was first equilibrated with ultrapure water (200 mL) before applying the sample. After washing with ultrapure water (60 mL) and 1 mM ammonium formate (25 mL), 2,7-anhydro-Neu5Ac was eluted with a gradient of ammonium formate ranging from 5 mM to 50 mM (150 mL) and freeze dried. After complete dryness, the powder was dissolved in ultrapure water (100 mL) and freeze dried again 3 times to remove volatile salts. 2,7-anhydro-Neu5Ac dissolved in ultrapure water (1.5 mL), centrifuged and filtered on a PTFE 0.45 μm membrane, was then desalted by BioGel P2 size exclusion chromatography. The obtained 2,7-anhydro-Neu5Ac was collected and freeze dried.

### 2.4 Synthesis of *N*-glycolylneuraminic acid-modified fetuin

Asialofetuin (25 mg/mL) was suspended in 100 mM Tris-HCl pH 8.5 with Neu5Gc (1.96 × 10^−5^ mol, 9.8 mM) and cytidine triphosphate (CTP) (1 eq., 9.8 mM) was added. After addition of a CMP-sialic acid synthetase from *Neisseria meningitidis* (1 UN, 0.5 UN/ml) and an α2–3-sialyltransferase from *Pasteurella multocida* (0.2 UN, 0.1 UN/mL), the reaction medium was incubated for 24 h at 37 °C under gentle shaking (115 rpm). The Neu5Gc disappearance as well as intermediate formation were monitored by thin-layer chromatography (TLC) using a 2:1:1 butanol/AcOH/water mobile phase (vol/vol/vol) and orcinol sugar stain (17 mM orcinol in 2:15:1 concentrated sulphuric acid/ethanol/water, vol/vol/vol). The reaction was stopped and the asialofetuin derivative was separated by Folch partitioning using a 2:1 chloroform/MeOH solution (vol/vol). The obtained precipitate was dried under nitrogen flux, re-suspended in 2 mL of ultrapure water and freeze dried. This product was then dialysed in 100 mM ammonium formate pH 6.5 for 4 h at 37 °C, 115 rpm, before performing the next step.

### 2.5 Analytical methods

For electrospray ionisation mass spectrometry (ESI-MS) analysis, 2,7-anhydro-Neu5Ac was dissolved in methanol (0.1 mg/mL) and filtered on a PTFE 0.45 μm membrane.

MS spectra were acquired on Expression CMS^L^ (Advion, Ithaca, USA) with ESI ionisation using direct injection operated in a negative ion mode. Advion Data Express (version 2.2.29.2) software package was used to evaluate the MS data.

For high resolution mass spectrometry analysis, the sample was diluted into 50% methanol/0.5% NH_4_OH and infused into a Synapt G2-Si mass spectrometer (Waters, Manchester, UK) at 5 μL/min using a Harvard Apparatus syringe pump. The mass spectrometer was controlled by Masslynx 4.1 software (Waters). It was operated in high resolution and negative ion mode and calibrated using sodium formate. The sample was analysed for 2 min with 1 s MS scan time over the range of 50–600 m/z with 3.0 kV capillary voltage, 40 V cone voltage, 100 °C cone temperature. Leu-enkephalin peptide (1 ng/μL, Waters) was infused at 10 μL/min as a lock mass (m/z 554.262) and measured every 10 s. Spectra were generated in Masslynx 4.1 by combining a number of scans, and peaks were centred using automatic peak detection with lock mass correction.

For Nuclear magnetic resonance (NMR) analysis, 2,7-anhydro-Neu5Ac (5 mg) or 2,7-anhydro-Neu5Gc (3 mg, crude) was dissolved in D_2_O (800 μL) and transferred into a 5 mm o.d. NMR tube for spectral acquisition. NMR spectra were run on a 600 MHz Bruker Avance III HD spectrometer (Bruker Biospin GmbH, Rheinstetten, Germany) fitted with a TCI cryoprobe and running Topspin 3.2 software. All experiments were performed at 300 K. ^1^H NMR spectra were acquired using the noesygppr1d pulse sequence for suppression of the residual water signal. Spectra were acquired with 32678 complex points in the time domain, spectral width 20.5 ppm, acquisition time 2.67 s, relaxation delay 3.0 s and 64 scans. Fourier transformation was carried out with zero filling to 65536 points and exponential multiplication to give line broadening of 0.3 Hz. A proton decoupled ^13^C spectrum of the same solution was acquired at 151 MHz using the zgpg30 pulse sequence with 32678 complex time domain points, spectral width 220.9 ppm, acquisition time 0.98 s, relaxation delay 2 s and 7200 scans. Fourier transformation was carried out with zero filling to 65536 points and exponential multiplication to give 3.0 Hz line broadening. The spectra were referenced using an external standard of methanol (δ_C_ = 51.43 ppm) in D_2_O. An HSQC spectrum of the same sample was recorded with the hsqcetgpprsisp2.2 pulse sequence with spectral widths of 12 ppm (^1^H) and 165 ppm (^13^C), a 2048 (t2) x 256 (t1) point acquisition matrix which was zero filled to 2048 × 1024 on 2D Fourier transformation, and 16 scans per t1 experiment. For complete and unambiguous assignment of the proton and carbon signal, we performed standard two-dimensional NMR techniques such as COSY and HSQC. ^13^C signals were assigned using the HSQC spectrum based on the known ^1^H chemical shifts.

For MS analysis of fetuin glycosylation, native asialofetuin or its derivative (100 μg) was dissolved in 100 μL ammonium bicarbonate (50 mM, pH 8) and heated to 100 °C for 5 min. After cooling the sample to room temperature, trypsin (20 μg) was added and the reaction was incubated at 37 °C for 16 h. Trypsin was heat inactivated at 100 °C for 5 min and PNGase F (8 IUB milliunits; New England Biolabs) was added to the mixture. The reaction was incubated at 37 °C for 16 h. The released glycans were purified using a C-18 Sep-Pak^®^ cartridges, prewet with 3 vol of methanol and equilibrated with 3 vol of 5% (vol/vol) acetic acid. Released glycans were eluted with 3 vol of 5% acetic acid and freeze-dried. The samples were dissolved in 200 μL of anhydrous DMSO, followed by the addition of ∼25 mg NaOH and 300 μL iodomethane. The permethylation reaction was incubated at room temperature for 60 min under vigorous shaking and quenched by the addition of 1 mL of 5% (vol/vol) acetic acid. The permethylated glycans were purified using a Hydrophilic-Lipophilic Balanced (HLB) copolymer Oasis cartridge (Waters, UK), prewet with 4 vol of methanol and equilibrated with 5% (vol/vol) methanol in H_2_O. Salts and other hydrophilic contaminants were washed with 5 vol of 5% methanol and permethylated glycans eluted with 3 vol of 100% methanol. The samples were dried under a gentle stream of nitrogen, dissolved in 10 μL of TA30 [30% (vol/vol) acetonitrile, 0.1% (vol/vol) trifluoroacetic acid] and mixed with equal amount of 2,5-dihydroxybenzoic acid (DHB; Sigma-Aldrich, UK; 10 mg/mL in TA30), before spotted onto an MTP 384 polished steel target plate (Bruker, UK). The samples were analysed by MALDI-TOF, using the Bruker Autoflex™ analyzer mass spectrometer (Applied Biosystems, Indiana, USA) in the positive-ion and reflectron mode.

For HPLC analysis of sialic acid content, asialofetuin and its sialylated derivatives were spiked with 50 ng KDN. Sialic acid and its derivatives were released by mild acid hydrolysis in acetic acid (2 N) at 80 °C for 3 h. The samples were dried using a Concentrator Plus (Eppendorf, UK) centrifugal evaporator and dissolved in 50 μL DMB labelling reagent [4,5-Methylenedioxy-1,2-phenylenediamine dihydrochloride (14 mM), sodium hydrosulfite (37 mM), acetic acid (2.8 M), β-mercaptoethanol (1.5 M)]. The labelling reaction was incubated at 50 °C for 2.5 h. The labelled samples were analysed by HPLC using a Luna C18(2) column (250 mm × 4.6 mm, 5 μm particle size and 100 Å pore size; Phenomenex, UK). Solvent A was acetonitrile, solvent B was methanol 50% vol/vol in H_2_O and solvent C was H_2_O. The gradient used was 4% A, 14% B and 82% C to 11% A, 14% B and 75% C, over 30 min. The excitation and emission wavelength was 373 and 448 nm, respectively. Elution times of labelled sialic acids were compared to known standards of Neu5Ac and Neu5Gc.

### 2.6 *In vitro* enzymatic activity assay

Substrates 3′SL or 4MU-Neu5Ac were incubated at 4 mg/mL with 1 nM *Rg*NanH in 100 mM ammonium formate for 24 h at 37 °C. The reaction was stopped by addition of an equal vol of ethanol prior to Folch partitioning. Neu5Gcα2-3Lac was incubated at 0.33 mg/mL with 60 nM *Rg*NanH in 20 mM sodium phosphate buffer (pH 6.5) containing 0.02 mg/mL bovine serum albumin (BSA) overnight at 37 °C. The reaction was stopped by boiling for 20 min, and the precipitated enzyme removed by centrifugation (17 000 *g*, 10 min, room temperature). In the case of all three substrates, a no-enzyme control was also carried out.

## 3 Results and discussion

### 3.1 Membrane-enclosed enzymatic synthesis of 2,7-anhydro-Neu5Ac

**Fig. 2:**
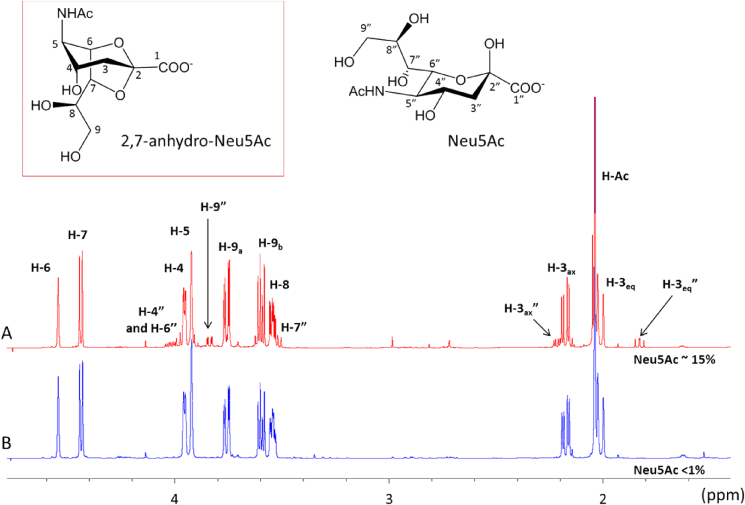
NMR spectra (600 MHz) of 2,7-anhydro-Neu5Ac obtained using a membrane enclosed synthesis with: (A) Fetuin (15 mg/mL) + *Rg*NanH (50 nM) (85% pure; 15% Neu5Ac); and (B) Fetuin (15 mg/mL) + *Rg*NanH (50 nM) + sialic acid aldolase (0.5 U/mL) (97% pure; <1% Neu5Ac).

Here we used the recombinant IT-sialidase (*Rg*NanH) from *R. gnavus* ATCC 29149 which produces 2,7-anhydro-Neu5Ac from α2,3-linked Neu5Ac substrates [13], and commercially available fetuin which contains about 8% in weight of α2,3-linked Neu5Ac for the enzymatic biosynthesis of 2,7-anhydro-Neu5Ac (Fig. 1). A Membrane Enclosed Enzymatic Catalysis (MEEC) approach [23] was used to achieve rapid and efficient recovery of the reaction product. This technique is based on the containment of the soluble enzyme in a dialysis membrane allowing the smaller product to diffuse through and be more readily recovered in the reaction buffer. Fetuin was chosen as a substrate as it is bulky and commercially available, allowing for both the enzyme (*Rg*NanH) and its substrate (fetuin) to be enclosed in the same dialysis membrane and therefore readily separated from the monosaccharide product [24]. The 2,7-anhydro-sialic acid derivative recovered in the reaction buffer was further purified by size exclusion chromatography on a Bio-gel P-2 column in order to remove the salts. ESI-MS and ^1^H and ^13^C NMR spectroscopy were used to monitor the purity of the reaction product. The mass spectrum and ^1^H NMR spectrum were identical to that of 2,7-anhydro-Neu5Ac obtained by methanolysis of Neu5Ac [19] or following enzymatic reaction of 4MU-Neu5Ac with the leech IT-sialidase [21] (Table 1; Fig. 2A, Fig. S1A). This process achieved 37% yield based on the 8% α2,3-linked sialic acid on fetuin and the product purity. Further analysis of the fetuin recovered after the synthesis revealed that its glycosylation pattern was similar to that of asialofetuin, attesting completion of the reaction (data not shown). However, the recovered product contained 85% 2,7-anhydro-Neu5Ac and 15% free sialic acid (Neu5Ac), as determined by ^1^H NMR (Fig. 2A). No Neu5Ac was detected in the control reaction in absence of *Rg*NanH, suggesting that no spontaneous degradation of fetuin occurred under the synthesis conditions or even after a prolonged period up to 50 h dialysis, as monitored by ESI-MS (data not shown). The use of alternative substrates such as 3′SL or 4MU-Neu5Ac did not affect the amount of free Neu5Ac produced (Fig. S2). Together these data indicated that Neu5Ac may be a by-product of the transglycosylation reaction.

**Table 1:** ^1^H and ^13^C NMR chemical shifts (ppm) of the 2,7-anhydro-sialic acid derivatives.

2,7-anhydro-Neu5Ac [21]										
	H-3ax	H-3eq	H-4	H-5	5-Ac	H-6	H-7	H-8	H-9	H-9′
H	2.17	2.01	3.92	3.95	2.04	4.54	4.43	3.54	3.76	3.59

**2,7-anhydro-Neu5Ac obtained with MEME synthesis**										
	H-3ax	H-3eq	H-4	H-5	5-Ac	H-6	H-7	H-8	H-9	H-9′
H	2.17	2.01	3.95	3.92	2.04	4.54	4.44	3.54	3.76	3.60
C	38.1	38.1	69.5	54.6	24.4	79.8	79.3	74.6	64.9	64.9

^**1**^**H and**^**13**^**C chemical shifts of 2,7-anhydro-Neu5Gc obtained with Neu5Gcα2-3Lac**										
	H-3ax	H-3eq	H-4	H-5	5-Gc	H-6	H-7	H-8	H-9	H-9′
H	2.22	2.03	3.99	4.02	4.15	4.58	4.48	3.57	3.78	3.60
C	38.2	38.2	69.7	54.6	63.6	80.0	79.6	75.0	65.2	65.2

**2,7-anhydro-Neu5Gc obtained with MEME synthesis**										
	H-3ax	H-3eq	H-4	H-5	5-Gc	H-6	H-7	H-8	H-9	H-9′
H	2.20	2.01	3.97	4.0	4.13	4.57	4.46	3.55	3.76	3.63
C	38.3	38.3	69.8	54.6	63.9	80.2	79.8	74.9	65.2	65.2

NMR solvent was D_2_O and temperature 300 K. Spectra were transformed using the Topspin NMR Suite software with a 0.3 Hz line broadening, and were manually phased, baseline corrected, and referenced by setting water signal to 4.77 ppm.

### 3.2 Coupling reaction of IT-sialidase and sialic acid aldolase

Considering the difficulty and the low efficiency of the separation of free Neu5Ac from 2,7-anhydro-Neu5Ac, a commercially available sialic acid aldolase from *E. coli* was introduced into the dialysis membrane with *Rg*NanH and fetuin. Sialic acid aldolases are efficient biocatalysts that convert free Neu5Ac into *N*-acetyl-mannosamine (ManNAc) and pyruvate [25]. Since 2,7-anhydro-Neu5Ac is resistant to degradation by sialic acid aldolase (data not shown), this enzyme was used to convert free sialic acid into smaller and uncharged enzymatic products which were easily eliminated using anion exchange chromatography on Dowex 1 × 8 resin (chloride form) prior to the size exclusion chromatography step. The 2,7-anhydro-Neu5Ac obtained during this one pot membrane-enclosed multienzyme (MEME) synthesis was about 96% pure, as shown by MS (Fig. S1B) and NMR (Fig. 2B), with a 33% yield. The Neu5Ac amount was reduced to traces representing less than 1%, and other impurities (3%) were identified as protein residues.

The multi-step enzymatic synthesis developed here is efficient, low cost and scalable to at least 20 mg, and allows the recovery of 2,7-anhydro-Neu5Ac with high purity. This method is also applicable to the synthesis of other 2,7-anhydro-sialic acid derivatives.

### 3.3 Membrane-enclosed enzymatic synthesis of 2,7-anhydro-Neu5Gc

**Fig. 3:**
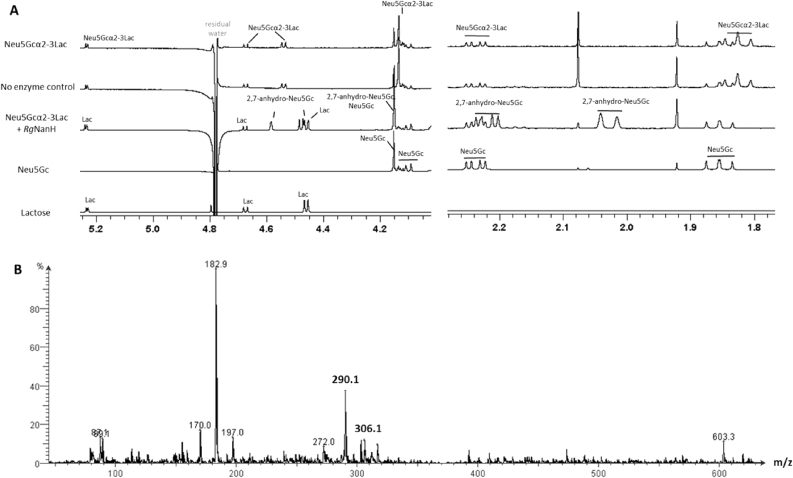
(A) NMR spectra (600 MHz) of MEME *Rg*NanH-catalysed reaction with standards of Lac, Neu5Gc and Neu5Gcα2-3Lac in overlay in the same buffer conditions (20 mM sodium phosphate, pH 6.5) without BSA. The control is the reaction incubated overnight in the same conditions as the MEME reaction (including BSA) but without enzyme present. (B) ESI(-)-MS spectra of the MEME *Rg*NanH-catalysed reaction with bovine submaxillary mucin without any purification step showing the production of 2,7-anhydro-Neu5Ac (m/z = 290.1) and 2,7-anhydro-Neu5Gc (m/z = 306.1).

The ability of the *Rg*NanH to produce 2,7-anhydro-Neu5Gc was first assessed using Neu5Gcα2-3Lac as substrate. Following an overnight incubation at 37 °C, the product of the reaction was analysed by NMR, showing the disappearance of the Neu5Gcα2-3Lac, the release of lactose and Neu5Gc, and the formation of a 2,7-anhydro compound similar to 2,7-anhydro-Neu5Ac by NMR (Fig. 3A). This 2,7-anhydro derivative was further identified as 2,7-anhydro-Neu5Gc by NMR (see Table 1) and by high resolution MS (HR-ESI-MS(-): m/z 1Calcd [C11H16NO9]-: 306.08304; found: 306.0831). In order to scale up the reaction, a membrane-enclosed synthesis was first performed using bovine submaxillary mucin as Neu5Gc donor, in place of fetuin which only displays a negligible amount of Neu5Gc [26]. Submaxillary mucin is 9–17% sialylated, and is the most abundant source of Neu5Gc among the commercially available glycoprotein, with Neu5Gc accounting for 15% of the total sialic acid content [27]. Although 2,7-anhydro-Neu5Gc was produced as monitored by ESI-MS (Fig. 3B), a large amount of 2,7-anhydro-Neu5Ac was also present, and the two 2,7-anhydro derivatives could not be efficiently separated. It is expected that using porcine submaxillary mucin as donor would greatly improve 2,7-anhydro-Neu5Gc yields as Neu5Gc represents 90% sialic acid [28], although the reaction product would still contain some 2,7-anhydro-Neu5Ac.

### 3.4 Synthesis of Neu5Gc-enriched glycoprotein

**Fig. 4:**
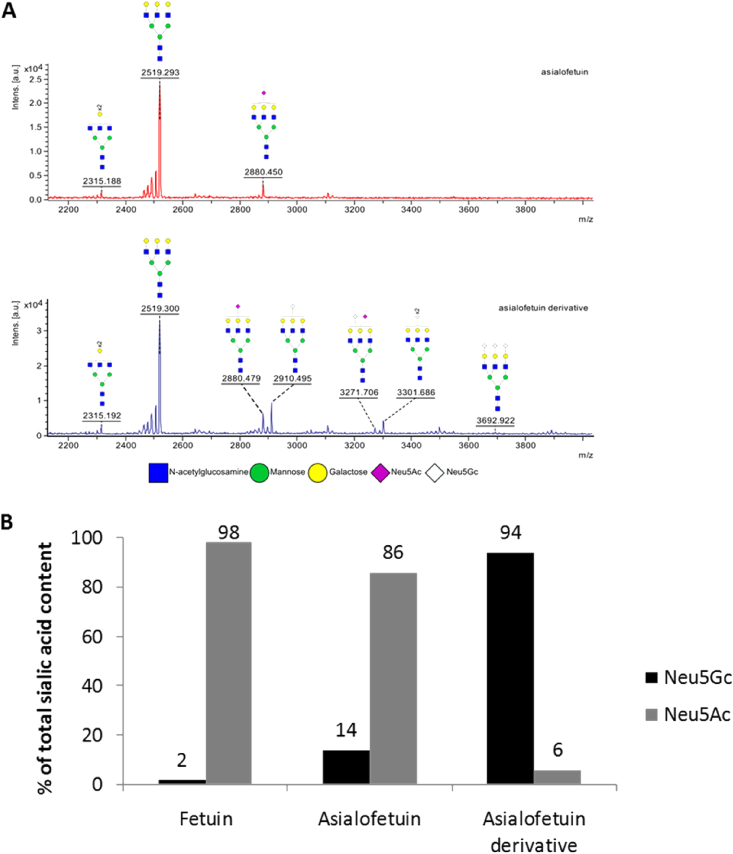
Analysis of the asialofetuin derivative after Neu5Gc enzymatic transfer. (A) Major oligosaccharides chains on the asialofetuin before (red) and after (blue) Neu5Gc enzymatic transfer characterised by MALDI-TOF; appearance of terminal Neu5Gc is observed after enzymatic treatment. (B) Neu5Ac and Neu5Gc relative amount on fetuin, asialofetuin and asialofetuin derivative after Neu5Gc transfer as quantified by HPLC after derivatization using 1,2-diamino-4,5-methylenedioxybenzene (DMB) reagent. (For interpretation of the references to colour in this figure legend, the reader is referred to the web version of this article.)

In the absence of a commercially available glycoprotein containing exclusively Neu5Gc, and to minimise 2,7-anhydro-Neu5Gc contamination with the Neu5Ac derivative, we attempted to transfer Neu5Gc onto an acceptor glycoprotein using a one-pot two-step enzymatic synthesis developed by Chen et al. [29], [30]. Asialofetuin was the most suitable acceptor as it contains ≤1.0% of Neu5Ac and the Neu5Ac free sites are good acceptor sites for the Neu5Gc transfer. We used a CMP-sialic acid synthase from *Neisseria meningitidis* group B to activate Neu5Gc to CMP-Neu5Gc, and a α2–3-sialyltransferase from *Pasteurella multocida* for the transfer of the activated sialic acid onto the acceptor glycoprotein with an α2,3-linkage. The obtained asialofetuin derivative was separated from the by-products by Folch partitioning and characterised by MALDI-TOF, showing the appearance of mono and di-sialylated oligosaccharide chains terminated with Neu5Gc (Fig. 4A). The relative amount of Neu5Gc and Neu5Ac on the asialofetuin derivative was further quantified by HPLC after derivatization with the 1,2-diamino-4,5-methylenedioxybenzene (DMB), using KDN as an internal response factor. The total sialic acid content ranged from 86% Neu5Ac and 14% Neu5Gc in asialofetuin to 6% Neu5Ac and 94% Neu5Gc after Neu5Gc transfer (Fig. 4B). This Neu5Gc-rich glycoprotein was then used in the membrane-enclosed enzymatic synthesis after dialysis in ammonium formate. The 2,7-anhydro-Neu5Gc obtained was characterised by NMR, and the profile found identical to the one previously obtained with Neu5Gcα2-3Lac as substrate (Table 1). The only difference between the 2,7-anhydro-Neu5Ac and the 2,7-anhydro-Neu5Gc is the absence of a signal for the acetyl group at 2.07 ppm on the 2,7-anhydro-Neu5Gc, replaced by a glycolyl one at 4.13 ppm.

In summary, we have developed a convenient and efficient membrane-enclosed multienzyme (MEME) approach for producing 2,7-anydro-modified sialic acids in a pure form, starting from readily available glycoproteins. The synthetic method reported herein offers general and straightforward access to a class of sialic acid derivatives recently discovered in the gut and shows promise to assess the biological significance and potential applications of 2,7-anydro-modified sialic acids in the context of drug discovery and biomedical research.
