# Supplementary material for: Membrane-enclosed multienzyme (MEME) synthesis of 2,7-anhydro-sialic acid derivatives
Source: Carbohydr Res. 2017 Nov 8;451:110–7. doi: 10.1016/j.carres.2017.08.008 (PMC5667892; doi:10.1016/j.carres.2017.08.008)
Supplement: Supplementary material [file mmc1.docx]

**Supplementary Figures**

**Figure S1:** ESI(-)-MS spectrum of the 2,7-anhydro-Neu5Ac obtained using a membrane enclosed synthesis with: (**A**) Fetuin (15 mg/mL) + *Rg*NanH (50nM), showing the presence of 2,7-anhydro-Neu5Ac (m/z = 289.5) and release of Neu5Ac (m/z = 307.6); (**B**) Fetuin (15 mg/mL) + *Rg*NanH (50 nM) + sialic acid aldolase (0.5 U/mL), showing the presence of only 2,7-anhydro-Neu5Ac (m/z = 290.1).


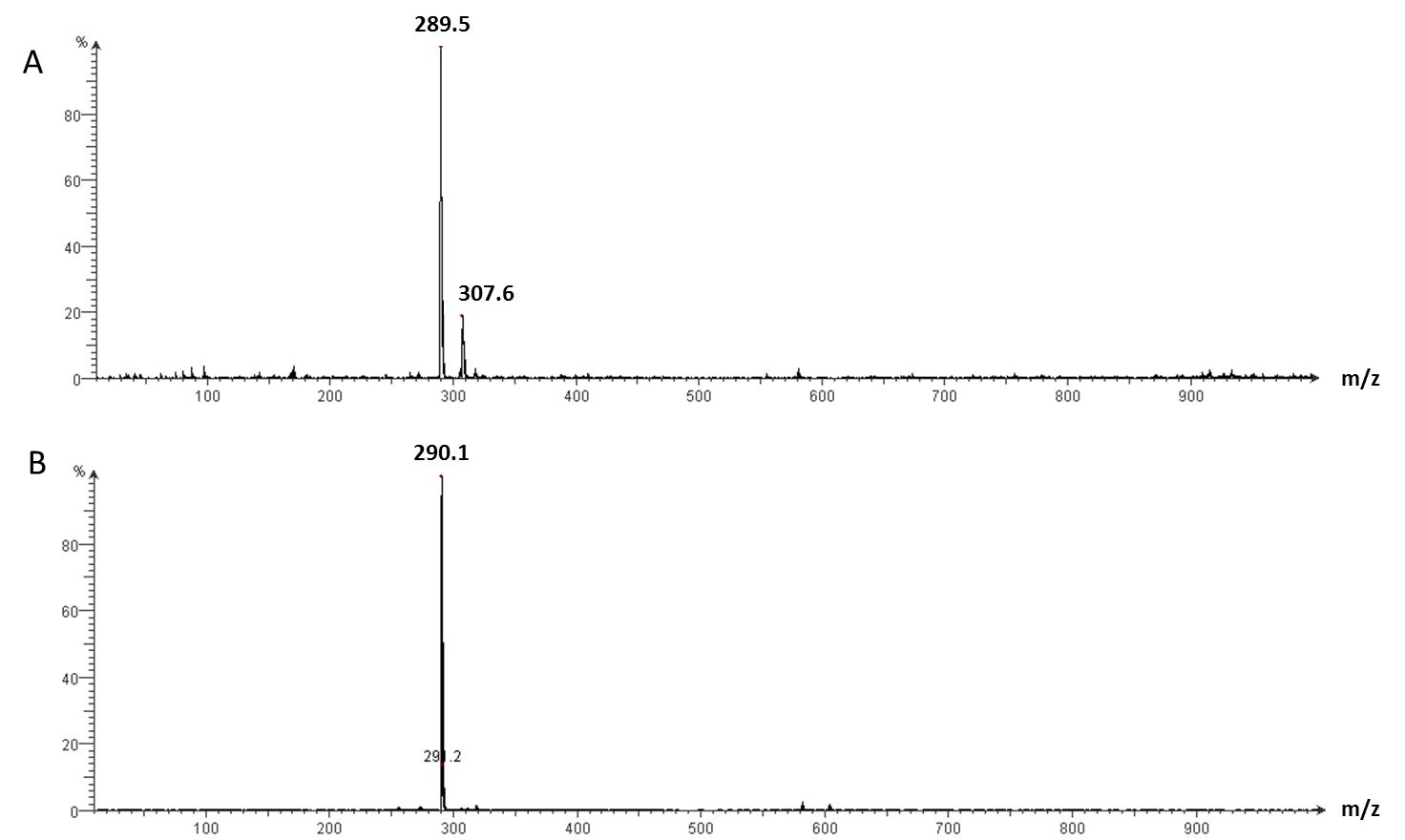


**Figure S2:** NMR analysis (600 MHz) of (**A**) Neu5Ac compared with the two *Rg*NanH-catalysed reactions with (**B**) 3’SL or (**C**) 4MU-Neu5Ac as substrates. In both cases, the amount of Neu5Ac released was about 15%.

**
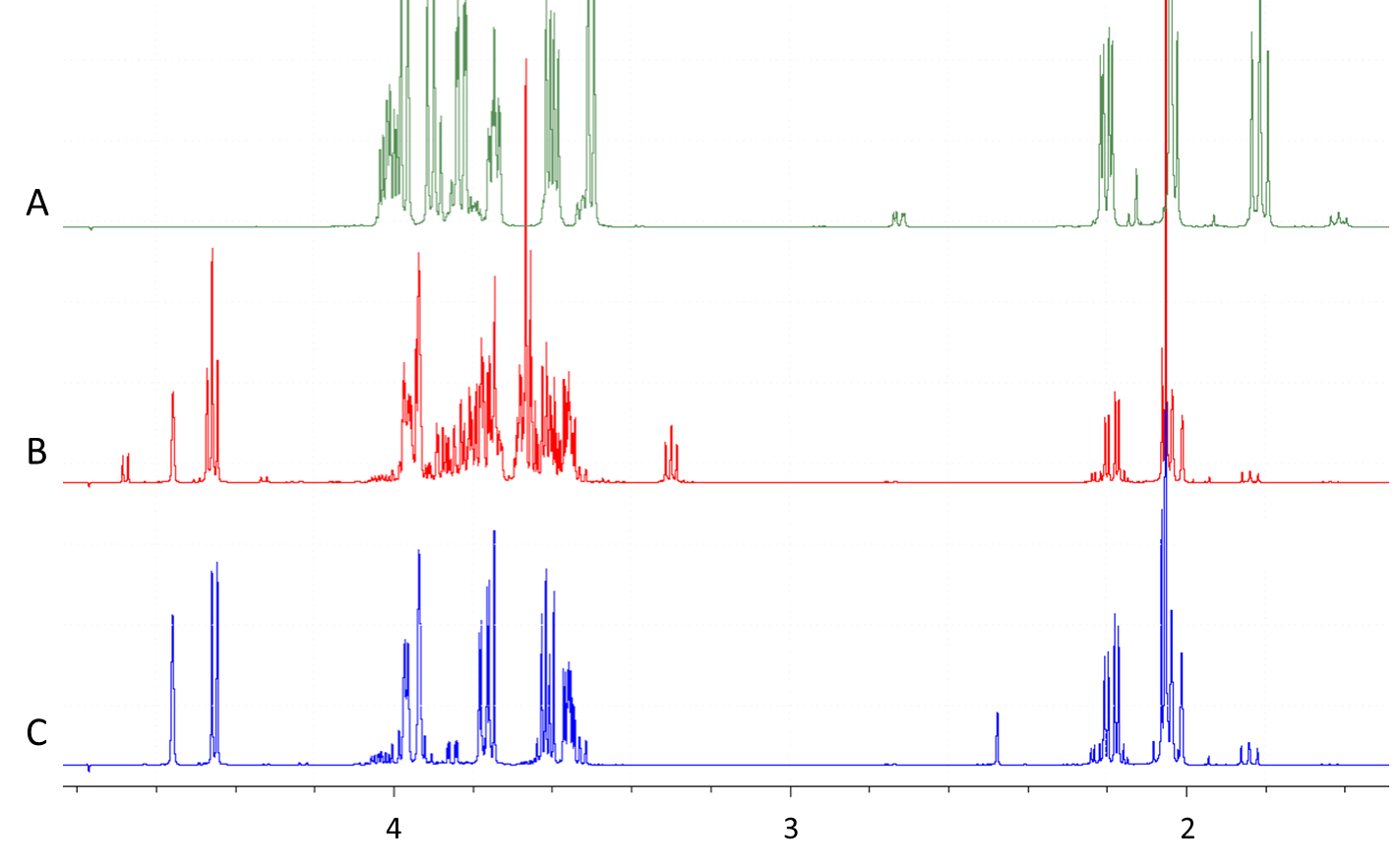
**
